# Asymmetry of sleep electrophysiological markers in patients with focal epilepsy

**DOI:** 10.1093/braincomms/fcad161

**Published:** 2023-05-24

**Authors:** Laurent Sheybani, Pierre Mégevand, Nicolas Roehri, Laurent Spinelli, Andreas Kleinschmidt, Pieter van Mierlo, Margitta Seeck, Serge Vulliémoz

**Affiliations:** EEG and Epileptology Unit, Neurology Service, Department of Clinical Neuroscience, Geneva University Hospitals, 1205 Geneva and Faculty of Medicine of Geneva, University of Geneva, 1206 Geneva, Switzerland; Epilepsy and Brain Networks Lab, Department of Clinical Neuroscience, Faculty of Medicine of Geneva, University of Geneva, 1206 Geneva, Switzerland; EEG and Epileptology Unit, Neurology Service, Department of Clinical Neuroscience, Geneva University Hospitals, 1205 Geneva and Faculty of Medicine of Geneva, University of Geneva, 1206 Geneva, Switzerland; Human Neuron Lab, Department of Fundamental Neuroscience, Faculty of Medicine of Geneva, University of Geneva, 1206 Geneva, Switzerland; EEG and Epileptology Unit, Neurology Service, Department of Clinical Neuroscience, Geneva University Hospitals, 1205 Geneva and Faculty of Medicine of Geneva, University of Geneva, 1206 Geneva, Switzerland; Epilepsy and Brain Networks Lab, Department of Clinical Neuroscience, Faculty of Medicine of Geneva, University of Geneva, 1206 Geneva, Switzerland; EEG and Epileptology Unit, Neurology Service, Department of Clinical Neuroscience, Geneva University Hospitals, 1205 Geneva and Faculty of Medicine of Geneva, University of Geneva, 1206 Geneva, Switzerland; Neurology Service, Department of Clinical neuroscience, Geneva University Hospitals, 1205 Geneva, Switzerland; Medical Image and Signal Processing (MEDISIP), Department of Electronics and Information Systems (ELIS), Ghent University, 9052 Ghent, Belgium; EEG and Epileptology Unit, Neurology Service, Department of Clinical Neuroscience, Geneva University Hospitals, 1205 Geneva and Faculty of Medicine of Geneva, University of Geneva, 1206 Geneva, Switzerland; EEG and Epileptology Unit, Neurology Service, Department of Clinical Neuroscience, Geneva University Hospitals, 1205 Geneva and Faculty of Medicine of Geneva, University of Geneva, 1206 Geneva, Switzerland; Epilepsy and Brain Networks Lab, Department of Clinical Neuroscience, Faculty of Medicine of Geneva, University of Geneva, 1206 Geneva, Switzerland

**Keywords:** epilepsy, sleep, electrophysiology

## Abstract

Sleep can modulate epileptic activities, but our knowledge of sleep perturbation by epilepsy remains sparse. Interestingly, epilepsy and sleep both present with defining electrophysiological features in the form of specific graphoelements on EEG. This raises the possibility to identify, within ongoing EEG activity, how epilepsy impacts and disrupts sleep. Here, we asked whether the presence of a lateralized epileptic focus interferes with the expression of the dominant electrophysiological hallmarks of sleep: slow oscillations, slow waves and spindles. To this aim, we conducted a cross-sectional study and analysed sleep recordings with surface EEG from 69 patients with focal epilepsy (age range at EEG: 17–61 years, 29 females, 34 left focal epilepsy). Comparing patients with left and right focal epilepsy, we assessed inter-hemispheric asymmetry of sleep slow oscillations power (delta range, 0.5–4 Hz); sleep slow wave density; amplitude, duration and slope; and spindle density, amplitude, duration as well as locking to slow oscillations. We found significantly different asymmetries in slow oscillation power (*P* < 0.01); slow wave amplitude (*P* < 0.05) and slope (*P* < 0.01); and spindle density (*P* < 0.0001) and amplitude (*P* < 0.05). To confirm that these population-based differences reflect actual patient-by-patient differences, we then tested whether asymmetry of sleep features can classify laterality of the epileptic focus using a decision tree and a 5-fold cross-validation. We show that classification accuracy is above chance level (accuracy of 65%, standard deviation: 5%) and significantly outperforms a classification based on a randomization of epileptic lateralization (randomization data accuracy: 50%, standard deviation 7%, unpaired *t*-test, *P* < 0.0001). Importantly, we show that classification of epileptic lateralization by the canonical epileptic biomarker, i.e. interictal epileptiform discharges, improves slightly but significantly when combined with electrophysiological hallmarks of physiological sleep (from 75% to 77%, *P* < 0.0001, one-way ANOVA + Sidak’s multiple comparisons test). Together, we establish that epilepsy is associated with inter-hemispheric perturbation of sleep-related activities and provide an in-depth multi-dimensional profile of the main sleep electrophysiological signatures in a large cohort of patients with focal epilepsy. We provide converging evidence that the underlying epileptic process interacts with the expression of sleep markers, in addition to triggering well-known pathological activities, such as interictal epileptiform discharges.

## Teaser

Sleep can modulate epilepsy, but we still lack a precise insight into how epilepsy affects sleep. Sheybani *et al*. show that sleep electrophysiological activities are asymmetrically expressed in patients with focal epilepsy, contributing to a better understanding of the interaction between epilepsy and normal brain network.

## Graphical Abstract

**Graphical abstract:**
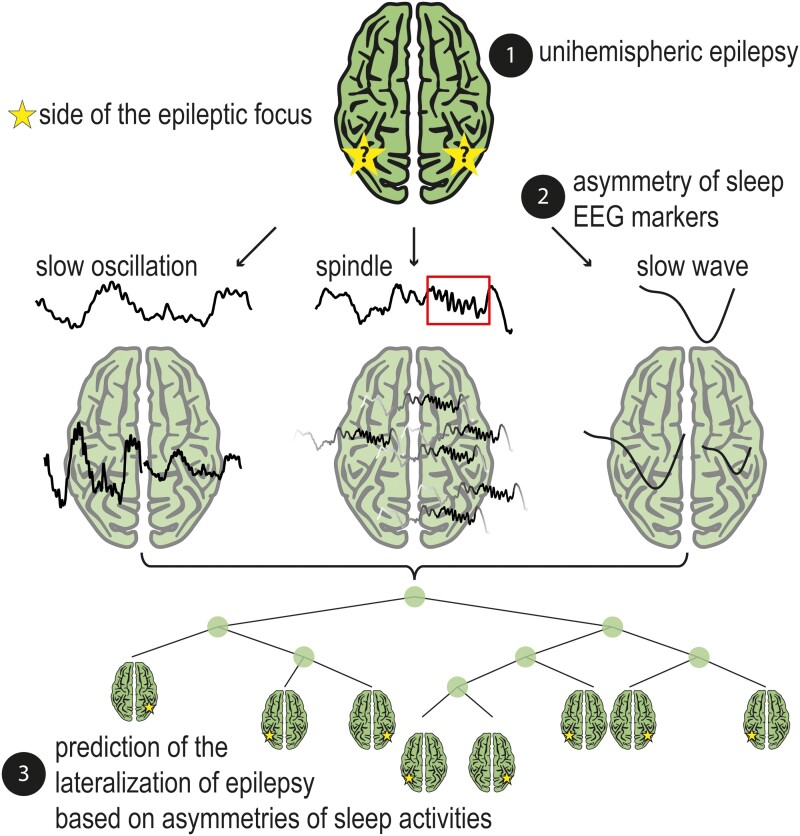

## Introduction

The effect of sleep on epileptic activity has been extensively studied.^1–8^ Increased seizure risk during sleep^6–8^ and reduced seizure risk with longer sleep duration^9^ are typical examples of how sleep modulates the expression of seizures on intermediate and slow time scales. On a more fine-grained time scale, there is also recent evidence that slow neural oscillations, typically seen during sleep, may modulate the probability of interictal epileptiform discharges (IEDs).^2,4,10–13^ Conversely but less studied, epilepsy also interferes with sleep or sleep-related activities,^14^ notably by perturbing sleep architecture or increasing the number of awakenings.^15,16^

Both epilepsy and sleep are associated with characteristic features on EEG, so that their interactions can be studied with this single investigational modality. However, only few studies have investigated how epileptic activity can perturb physiological brain activity during sleep. Sleep spindle density (occurrence per minute), an index of physiological sleep, has generally been found to be reduced in patients with focal epilepsy,^17–20^ although one study has also observed an increase in the epileptic hemisphere.^21^ Another such index is slow wave sleep activity. Its proxy, spectral power in the delta-band, is enhanced in patients with epilepsy (right, left or bilateral foci).^22–25^ This effect tends to be lateralized to the epileptic hemisphere and could at first glance be considered pathological slowing related to the epileptic focus.^22–25^ However, this increased slow wave activity displays typical features of normal sleep homeostasis, and in particular a progressive attenuation during non-rapid eye movement (NREM) stages along the night,^23^ suggesting that it reflects a physiological homeostatic mechanism and not mere epilepsy-related pathological activity.

Specific features of physiological sleep, as slow wave activity, can present inter-hemispheric asymmetries.^26^ Strong evidence, including intracranial recording,^27–30^ supports the fact that the modulation of specific sleep components by physiological processes is typically local.^26,31–38^ In healthy subjects, localized decrease in delta power during sleep has been described specifically in the left hemispheric default-mode network.^26^ This effect is limited to a first night of sleeping in a novel environment and believed to be due to increased vigilance under these conditions.^26^ Accordingly, in patients with left focal epilepsy, one would predict that the homeostatic^23^ and vigilance^26^ mechanisms drive two opposed and competing effects on delta activity in the left, epileptic hemisphere during a first night of sleep recording. Together, focal epilepsy could interfere with the expression of sleep features, resulting in uneven modulations across hemispheres. Probing the perturbation of sleep-related activities by epilepsy hence requires dissociating the analysis of both hemispheres and relating it to the lateralization of epileptic activity. Moreover, it also requires recording and analysing the stability of these markers across at least two nights.

To improve our understanding of the impact of an epileptic focus on physiological sleep, we conceived the present study taking into account the aforementioned incongruencies and potential influences identified in previous studies on epilepsy and sleep. We used scalp EEG recordings in patients with focal epilepsy during sleep to address whether a lateralized epileptic focus is associated with inter-hemispheric asymmetries of cardinal physiological sleep features (slow oscillations, sleep slow waves and spindles). We further addressed whether any effects were stable across two consecutive nights. After identifying differences between the populations of left and right focal epilepsy, we used a classifier (decision tree) to confirm that these population-based differences are verified in a patient-by-patient analysis. Ultimately, we tested whether the asymmetry of physiological sleep features can improve the classification of epileptic lateralization beyond what is attributable to IEDs alone. Our work provides an in-depth analysis of the main electrophysiological sleep features in a large cohort of patients with focal epilepsy. We establish that sleep features and IEDs are comparatively shaped by the same underlying pathological process, thus establishing sleep indices as a mirroring biomarker of focal epilepsy.

## Materials and methods

### Patient selection

We screened patients who underwent long-term (≥ 2 nights) scalp EEG recording for refractory epilepsy at the Geneva University Hospitals between January 2017 and March 2021. We applied the following inclusion criteria: (i) age ≥ 16 years, (ii) disease onset ≥ 1 year, (iii) lateralized seizure-onset zone based on best clinical evidence (i.e. converging clinico-radiological evidence, interictal 24 h EEG and MRI) and (iv) no previous neurosurgical operation. Patients with suspected bilateral, unknown lateralization or discordance between clinical, radiological and neuropsychological evidence that impeded lateralization were thus not included. Exclusion criteria included: (i) seizure occurring during analysed sleep, (ii) no epileptic disorder confirmed, (iii) missing data (e.g. recording < 20 min of NREM 2 and 3), (iv) unreliable scoring (e.g. interictal activity too intense) and (v) medically requested sleep deprivation. Study size was determined by availability of data. All eligible patients on the basis of these criteria were included. The study was performed in accordance with the Commission Cantonale d’Ethique de la Recherche, Canton de Genève, Switzerland (protocol #2020-00331).

### Data acquisition and pre-processing

**Figure 1:**
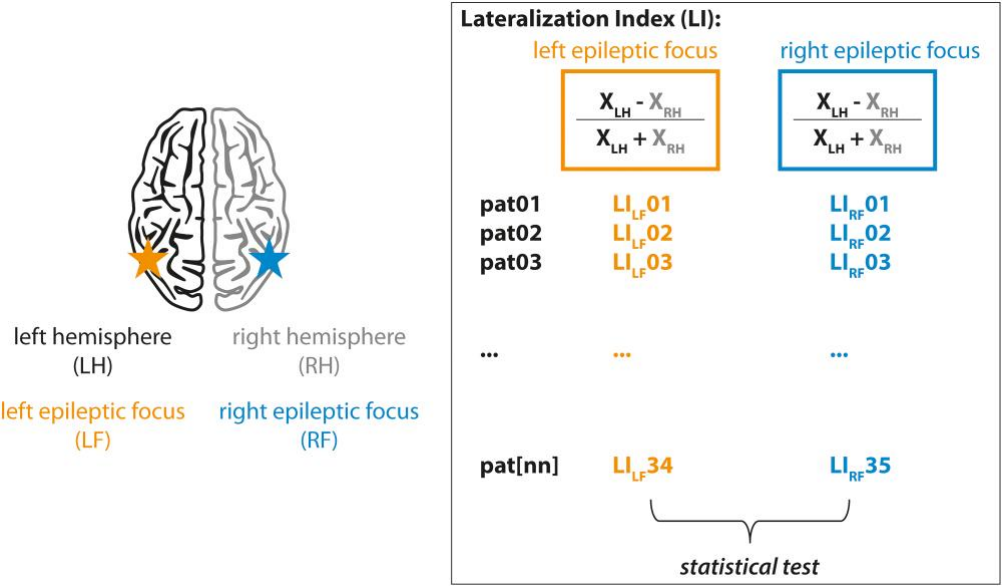
**Methodological strategy.** We used the LI for most of our statistical analyses for reasons explained in the ‘Materials and methods’ section, as well as in the ‘Discussion’ section. We categorized patients with left (orange, left part of the box) versus right (blue, right part of the box) epileptic focus based on best available clinical information (at least clinical semiology, interictal 24 h EEG and MRI). The same colour code applies to all figures in the paper. Within each group (left and right epileptic focus) and each patient, we measure the LI, which corresponds to the difference of each individual sleep feature noted ‘X’ (see below) between left minus right hemisphere (≠ epileptic hemisphere) divided by the sum of X between left plus right hemisphere. We obtain one value per patient and ultimately process a statistical test comparing patients with left versus right epileptic focus. X indicates slow oscillations power; slow wave amplitude, duration, density and slope; and spindle density, amplitude, duration and grouping to slow oscillations. There are 34 values for each sleep feature for the left focal epilepsy group and 35 for the right focal epilepsy group. pat = patient; LF = left focal epilepsy; RF = right focal epilepsy; LH = left hemisphere; RH = right hemisphere.

Patients were recorded with a Micromed EEG system (sampling frequency for acquisition: 256 Hz, high-pass: 0.15 Hz, no low-pass), using 25–38 electrodes according to the current international recommendations.^39^ Each montage consisted in a classical extended longitudinal bipolar montage with inferior temporal electrodes (‘Fp2-F10’, ‘F10-FT10’, ‘FT10-P10’, ‘P10-O2’, ‘Fp2-F8’, ‘F8-T8’, ‘T8-P8’, ‘P8-O2’, ‘Fp2-F4’, ‘F4-C4’, ‘C4-P4’, ‘P4-O2’, ‘Fp1-F3’, ‘F3-C3’, ‘C3-P3’, ‘P3-O1’, ‘Fp1-F7’, ‘F7-T7’, ‘T7-P7’, ‘P7-O1’, ‘Fp1-F9’, ‘F9-FT9’, ‘FT9-P9’, ‘P9-O1’). Specific electrodes were chosen for identifying sleep-related graphoelements (see below). We identified NREM stages 2 and 3 based on conventional criteria,^40^ after excluding periods with artefacts. We extracted ≥ 20 min of NREM 2 and ≥ 20 min of NREM 3. In preliminary analyses of intra-individual stability of sleep marker asymmetry across nights, we analysed the first two consecutive nights of long-term EEG monitoring. As within the left and right epilepsy groups no significant difference across the two nights was observed in any of the sleep activities studied, we pooled together both nights by calculating the average per patient for each sleep feature. All results are based on these averages, except for the classifier, which was based on the first night only for the sake of clinical applicability. All signal analyses were performed in the Matlab® (MathWorks) environment (see below for specific functions, including Fieldtrip^41^). Our strategy for statistical analysis is further discussed below and in Fig. 1.

### Identification and processing of slow oscillations

**Figure 2:**
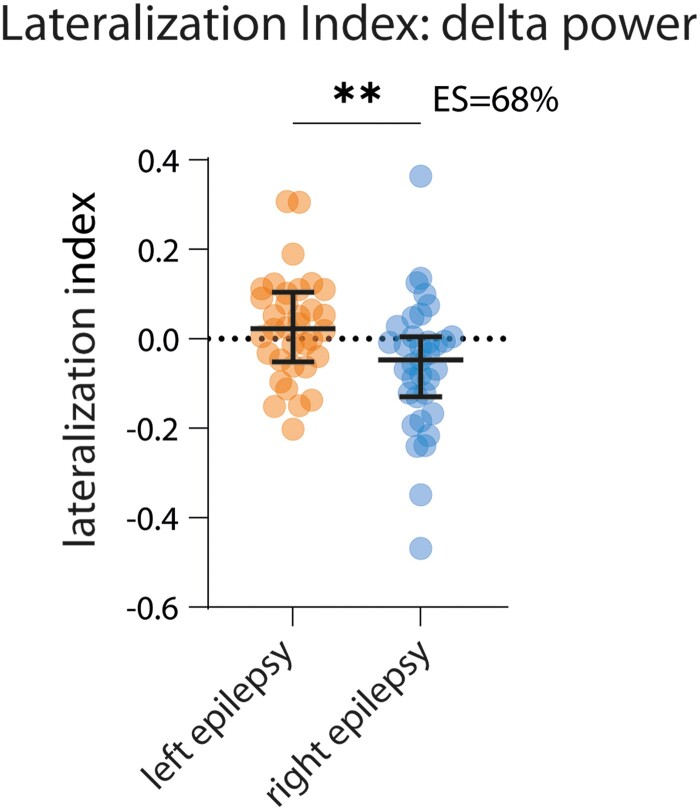
**Delta activity is asymmetrical during sleep in patients with focal epilepsy.** LI is significantly different between patients with left (orange, left-sided dots, *n* = 34 patients) and right (blue, right-sided dots, *n* = 35 patients) focal epilepsy (Mann–Whitney test, **P = 0.0081). Bars indicate median ± IQ. ES = non-parametric effect size (see ‘Materials and methods’ section).

Slow oscillations in ongoing activity are assessed by measuring spectral power over several seconds to minutes. Power in the delta frequency range (0.5–4 Hz) was used as a proxy of slow oscillations and was identified via the Welch’s method (*pwelch.m* function of Matlab®) during NREM 2 and NREM 3. Since we were interested in inter-hemispheric asymmetries (Fig. 2), the bipolar montage was chosen to maximize the identification of focal activities. All ‘lateralized’ electrode pairs of the longitudinal montage were included, i.e. excluding midline electrodes.

### Identification and processing of slow waves

**Figure 3:**
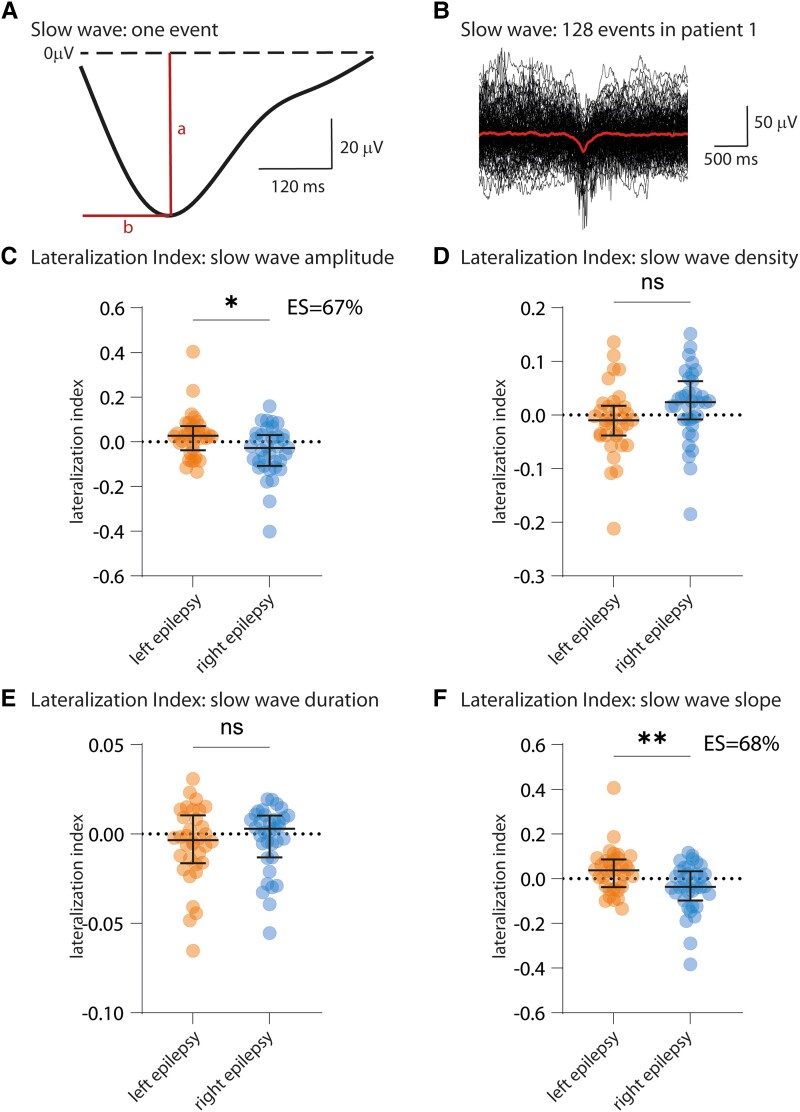
**Slow wave amplitude and slope are asymmetrical in patients with focal epilepsy. (A)** Example of a sleep slow wave truncated at onset (positive-to-negative 0-crossing) and offset (negative-to-positive 0-crossing). The amplitude (a) is the absolute value of the minimum and the duration is the timing between the positive-to-negative 0-crossing to negative-to-positive 0-crossing. The slope is the ratio between the amplitude and timing to minimum (b). **(B)** Illustration of 128 slow waves identified in the left hemisphere of subject 1 (red: mean activity). **(C)** The LI of slow wave amplitude is significantly different between patients with left and right focal epilepsy (*P = 0.0158). **(D** and **E)** No significant difference of LI is identified for slow wave density and duration. **(F)** The LI of slow wave slope is significantly different between left and right focal epilepsies (**P = 0.0096). Bars indicate median ± IQ. Statistical test: Kruskal–Wallis + Dunn’s multiple comparisons test. Orange, left-sided dots: *n* = 34 patients with left focal epilepsy. Blue, right-sided dots: *n* = 35 patients with right focal epilepsy. ES = non-parametric effect size (see ‘Materials and methods’ section).

After the first analysis of slow oscillations via continuous spectral power in the range of minutes, we next considered sleep slow waves as discrete events (Fig. 3A and B).^42^ Sleep slow waves were identified during NREM 2 and 3, following criteria described in the literature^4,43^ as negative potentials lasting 0.25–1 s after filtering the raw data between 0.5 and 4 Hz (Butterworth filter). In line with the identification of delta power, we used longitudinal bipolar traces (F3-C3, F7-T7, F4-C4, F8-T8) to identify local activities. Since slow waves are defined as negative potentials, we conserved slow waves identified in the bipolar montage only if a slow wave was also identified as negative potential in the average referenced signal (see Supplementary Materials and Methods and Supplementary Fig. 1).

Slow wave amplitude was defined as the absolute value of the slow wave minimum (letter ‘a’ in Fig. 3A); duration as the time between the slow wave positive-to-negative and negative-to-positive zero crossing (dashed line in Fig. 3A); density as the occurrence of slow waves per minute; and slope as the ratio between amplitude and timing to minimum (‘a’ divided by ‘b’ in Fig. 3A).

### Spindles identification and processing

Spindles were identified only during NREM 2, as they mainly occur during that stage.^44^ Spindles were automatically identified in bipolar channels (‘C3-FT9’ for left and ‘C4-FT10’ for right spindles) using a published detector^45^ implemented in the toolbox Wonambi (https://wonambi-python.github.io/). We calculated spindle density (occurrence per minute), amplitude, duration and locking. Of note, benzodiazepines can induce fast rhythms in the beta range, which could be interpreted as spindles, but benzodiazepine usage was similar for patients with left and right focal epilepsies (Table 1).

**Table 1:** Population statistics

	Right epilepsy	Left epilepsy	*P*-value	Test
Female	49%	35%	*0*.*33*	Fisher’s
Duration of analysed data (first night), hours	4.5	4.4	*>0*.*99*	Kruskal–Wallis
Duration of analysed data (second night), hours	3.9	3.5	*0*.*31*	Kruskal–Wallis
Number of seizures during day 1 or 2^a^	2	4	*0*.*68*	Fisher’s
Age at onset: median (IQ), years	18 (12–27)	16 (8–24)	*0*.*29*	Mann–Whitney
Disease duration: median (IQ), years	9 (4–27)	13 (6–22)	*0*.*56*	Mann–Whitney
Age at EEG: median (IQ), years	34 (24–47)	33 (22–39)	*0*.*44*	Mann–Whitney
AEDs: median (IQ)	2 (2–3)	2 (1–3)	*0*.*37*	Mann–Whitney
BZD: median (IQ)	0 (0–1)	0 (0–0)	*0*.*51*	Mann–Whitney
Temporal involvement	66%	76%	*0*.*43*	Fisher’s
Non-lesional	46%	44%	*>0*.*99*	Fisher’s
Right-handed	71%	88%	*0*.*13*	Fisher’s
SAS	11%	6%	*0*.*67*	Fisher’s

Population statistics in patients with right versus left focal epilepsy. Potential confounding factors were represented equally in both populations.

a Patients who were not included during night 2 are not included in this analysis.

AED = anti-epileptic drugs; BZD = benzodiazepine; SAS = sleep apnoea syndrome.

To assess spindle amplitude, epochs of ±3 s around the maximal trough of each spindle were filtered between 11 and 16 Hz, and the amplitude was measured as the range between individual spindle maxima and minima.^44^ Spindle duration was calculated as the difference between the offset and onset of each individual spindle, which was provided by the automated detector^45^ (see Supplementary Fig. 2A and B for examples of detected spindles and Supplementary Fig. 2C for the time–frequency representation of 767 spindles identified in patient #4 using the function *spectrogram.m* from Matlab). To obtain the time–frequency decomposition depicted in Supplementary Fig. 2C, we normalized the power to the one of phase shuffled data (using *phaseshuffle.m*^46^). The spectrogram on the right is the oscillatory component of the data (original spectrogram minus the 1/f component of the original data) between −0.5 and 0.5 s around the middle of each individual spindles’ time window using the Fieldtrip code *ft_freqanalysis.m*,^41^ method ‘irasa’. This figure was intended for illustration only. To assess grouping (i.e. phase-locking) of spindles to slow oscillations, we used the timepoint of maximal trough of each spindle (in the data filtered between 11 and 16 Hz). We then filtered the same (raw) epochs between 0.5 and 4 Hz and obtained the instantaneous phase of the Hilbert-transformed signal (*hilbert.m* function, Matlab®). We then calculated inter-trial coherence (ITC)^11,47^ across epochs, locked to the maximal through of spindles to assess locking to slow oscillations. The ITC is defined by:


$$\mathrm{ITC}=\;\frac{1}{N}\cdot \left|\overset{N}{\underset{k=1}{\sum }}\;e^{i\cdot \varphi }\right|$$

where *N* is the number of epochs, *k* is the current epoch and *φ* is the phase of the signal. To study spindles grouping to slow waves, we used the ITC rather than phase–amplitude coupling because the latter is affected by spindles amplitude. Since spindles amplitude was a parameter that was analysed in this study (and furthermore asymmetrical), we did not want it to affect the measure of spindles grouping, explaining why we did not use phase–amplitude coupling.

### Statistical analysis

Most statistical analyses were performed on the lateralization index (LI, see Fig. 1 for our methodological strategy),^26^ which is defined by:


$$LI=\frac{X_{\mathrm{left}}-X_{\mathrm{right}}}{X_{\mathrm{left}}+X_{\mathrm{right}}}$$

where *X* is one of our measured variables in one distinct hemisphere: either delta power; slow wave density, amplitude, duration or slope; or spindle density, amplitude, duration or locking to slow oscillations. We used the LI, rather than raw variables of the left against right hemispheres, because we reasoned that the median of the differences is more accurate than the difference of the medians (especially when there is variability across subjects, see ‘Discussion’ section). To assess statistical significance of LI differences, we used non-parametric tests (Mann–Whitney test for delta power and Kruskal–Wallis test + Dunn’s correction for multiple comparisons for slow waves and spindles). *P*-values mentioned in the manuscript are adjusted for multiple comparisons.

**Figure 4:**
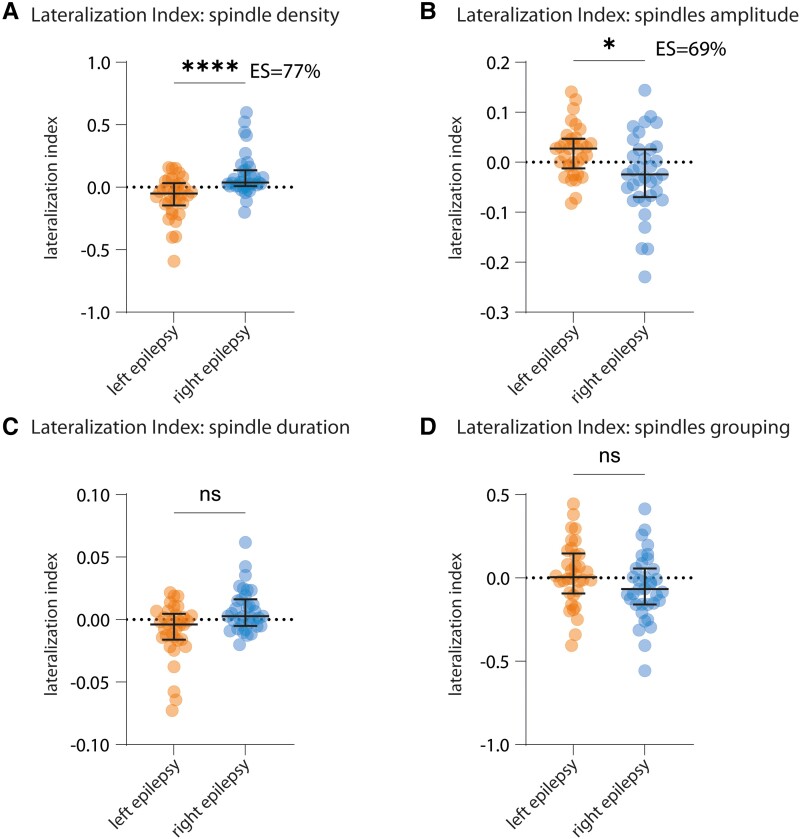
**Spindles density and amplitude are asymmetrical in patients with focal epilepsy. (A)** LI of spindle density between patients with left (orange, left-sided dots) and right (blue, right-sided dots) focal epilepsy is significantly different and in favour of the non-epileptogenic hemisphere (****P < 0.0001). **(B)** The LI of spindle amplitude is significantly different and in favour of the epileptogenic hemisphere (*P = 0.0402). **(C** and **D)** The LI of spindles duration and locking to slow oscillations is similar in both groups. Bars indicate median ± IQ. Statistical test: Kruskal–Wallis + Dunn’s multiple comparisons test. Orange, left-sided dots: *n* = 34 patients with left focal epilepsy. Blue, right-sided dots: *n* = 35 patients with right focal epilepsy. ES = non-parametric effect size (see ‘Materials and methods’ section).

All statistical analyses were performed with Prism GraphPad®. In Figs. 2–4, we provide also the effect size using a non-parametric estimator called the probability of superiority,^48^ which is defined by:


$$ES=\frac{\left[\#(x_{\mathrm{left}}>x_{\mathrm{right}})+0.5\cdot \#(x_{\mathrm{left}}=x_{\mathrm{right}})\right]}{\mathrm{length}(x_{\mathrm{left}})\cdot \mathrm{length}(x_{\mathrm{right}})}$$

where *x*_left_ and *x*_right_ are the values being compared (LI in left and right focal epilepsy), # is the count function and *ES* the effect size. This value provides the probability that a randomly selected variable within *x*_left_ will be higher (or respectively lower, if the LI is negative) than a randomly selected variable within *x*_right_.^48^ Details of the statistical results can be found in Supplementary Table 2.

### Classification based on sleep features

We used a decision tree with all nine sleep activity features as predictors: slow oscillations; slow wave amplitude, density, duration and slope; and spindle density, amplitude, duration and locking. To avoid bias from the results of the univariate analyses, we did not constrain the classifier to use those features that showed significant asymmetry differences between right and left focal epilepsy. We used a tree classifier (*fitctree.m*), including a 5-fold cross-validation step. Cross-validation is a crucial step in classification to prevent overfitting (or underfitting). It divides the dataset into five subsets, trains the classifier on all but the set-aside patients and then tests the accuracy (% of rightly classified patients) on the set-aside patients. This operation is repeated five times for each of the five subsets. Hence, the validation step records the accuracy on a subset of patients that is independent and unknown to the trained classifier. We repeated these operations 5000 times and obtained, at each iteration, a mean accuracy, a mean sensitivity, specificity, positive predictive value and negative predictive value. Furthermore, the 5000 loops allow estimating the error of the accuracy (standard deviation, SD). We then compared accuracy with that from 5000 iterations of classification based on the same data, but with lateralization shuffled at each iteration for each patient. Last, we used Fisher’s exact test to assess significance of classification based on the contingency table (2 × 2 table with true positive, true negative, false positive and false negative).

**Figure 5:**
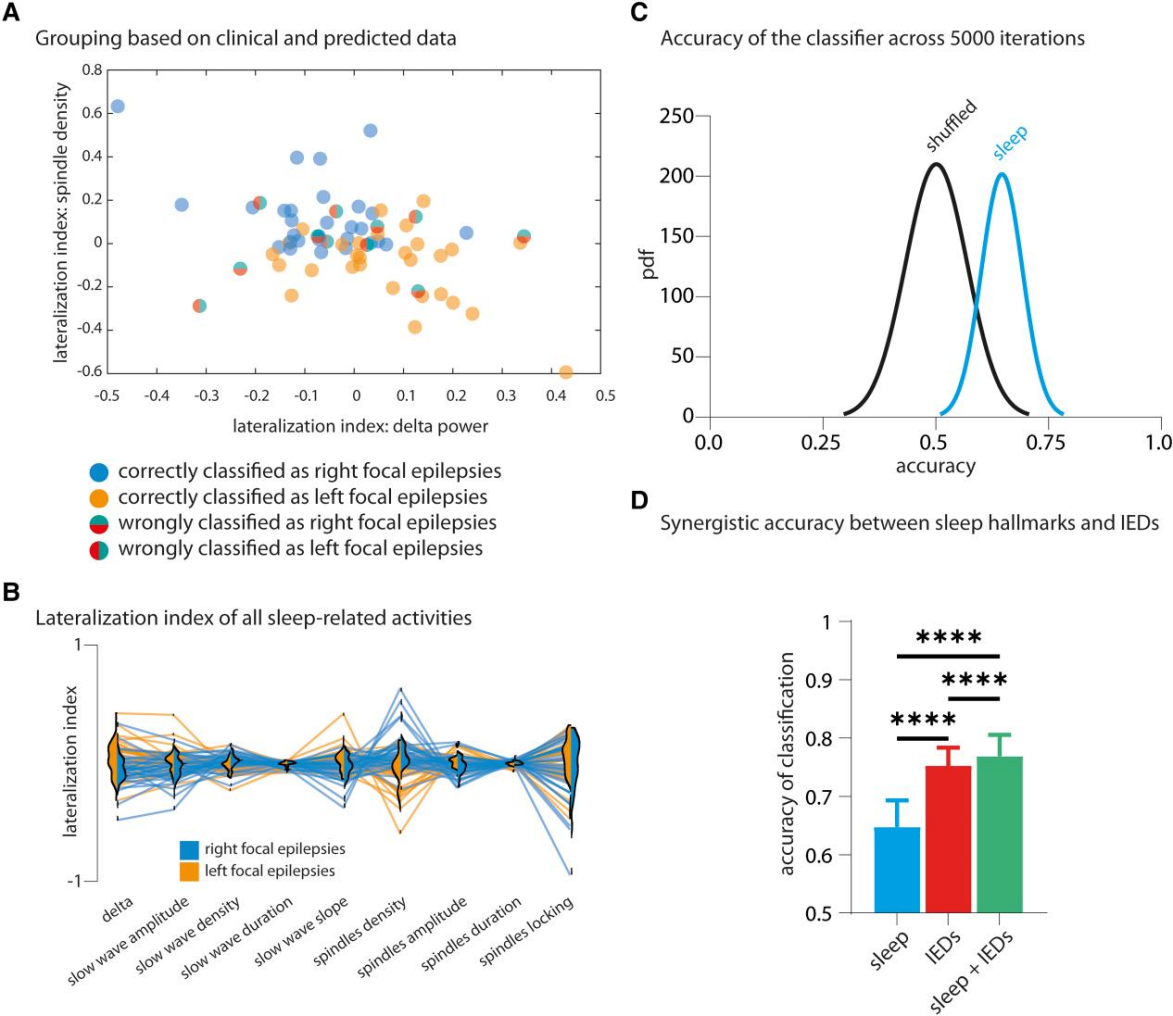
**Classification of the lateralization of epilepsy using sleep features.** For the sake of clarity, we illustrate in panel **A** the efficiency of the classifier using only two dimensions (delta power and spindle density). **(A)** Distribution of the 68 subjects based on the LI of delta power during first night (*x*-axis) and LI of spindle density during first night (*y*-axis). Filled dots are correctly classified patients. Split-coloured dots are wrongly classified patients. **(B)** Parallel plot of the classifier with violin plots overlapping to show a comprehensive representation of all sleep features analysed in the study. The *y*-axis is the LI of respective sleep features, indicated on the *x*-axis. In the parallel plot, each line represents one patient (left epilepsy in orange and right epilepsy in blue). The violin plots show the probability distribution of respective LI. **(C)** Probability density function of accuracy values across 5000 iterations of classification. Sleep data (blue, right-sided curve): the classification is based on 68 patients, without manipulation. Shuffled data (black, left-sided curve): the classification is based on 68 patients in whom the lateralization of epilepsy is shuffled (the number of right and left focal epilepsy does not change, and the individual values of each LI do not change). **(D)** There is a synergetic improvement in the accuracy of the epileptic lateralization using sleep hallmarks and IEDs LI (ANOVA + Sidak’s multiple comparisons test, ****P < 0.0001). Accuracy using sleep hallmarks only (sleep), IEDs only (IEDs) and sleep activities and IEDs (sleep + IEDs). Data are mean + SD (across *n* = 5000 loops of classification).

To test the added value of sleep-related EEG parameters to IEDs in the classification of left and right focal epilepsy, we used an ANOVA + Sidak’s multiple comparisons tests to compare the accuracy using the LI of sleep graphoelements only, the LI of IEDs only (see Supplementary Materials and Methods) and the LI of both sleep graphoelements and IEDs. For the sake of potential clinical applicability, we only used data from night 1 for the decision tree. The ‘importance’ of each predictor was determined using the Matlab function *predictorImportance.m*. In brief, this function calculates, for each predictor, the change in the probability of wrong classification at each node (i.e. the change in the classification performance provided by each predictor at each node) and divides it by the number of branch nodes. An estimate of 0 denotes the smallest importance and 1 highest importance. A comprehensive overview of the different sleep activity features can be seen in the parallel plot in Fig. 5B. Note that the use of LI, rather than raw data, normalizes the data, which prevents the classifier from being biased towards features with high values (e.g. the ITC goes from 0 to 1, while the amplitude of slow waves is in the range of tens, which could thus weaken the potential contribution of the ITC to the classifier).

## Results

### Patient characteristics

We included 69 patients (29 females) who underwent long-term (≥ 2 nights) evaluation of right (*n* = 35) and left (*n* = 34) focal epilepsy (Table 1; Supplementary Table 1). Median age at EEG was 33 years (interquartile range, IQ: 23–42 years) and median disease duration 12 years (IQ: 5–23 years). Most cases suffered from temporal (*n* = 32) or frontal lobe epilepsy (*n* = 11). Anatomopathological examination and imaging assessments revealed a non-lesional aetiology in most cases (*n* = 31, 45%) followed by hippocampal sclerosis (*n* = 15, 22%). The median number of anti-epileptic drugs per patient was 2 in right and left focal epilepsies, which was not significantly different (Table 1). There were no significant differences between the two groups in benzodiazepine usage, duration of analysed data, number of seizures during day 1 or 2, age at disease onset, disease duration, hand laterality or known sleep apnoea syndrome (Table 1).

### Sleep-specific EEG features are asymmetric in patients with focal epilepsy

We observed a significantly different delta power LI for patients with left and right focal epilepsy, with higher delta power in the epileptic than in the non-epileptic hemisphere (**P = 0.0081, Fig. 2; Supplementary Table 2). In line with this, we also observed a significantly different LI of slow wave amplitude for patients with left and right focal epilepsy, with higher amplitude in the epileptic hemisphere (*P = 0.0158, Fig. 3C; Supplementary Table 2). The LI of slow wave slope was also significantly different, showing a steeper slope in the epileptic hemisphere (**P = 0.0096, Fig. 3F, Supplementary Table 2, respectively). Of note, time–frequency analysis of slow wave onset did not show the typical time–frequency pattern of IEDs, confirming that intercurrent IEDs do not account for the observed results (Supplementary Fig. 3 and Supplementary Materials and Methods).

For spindles, we observed a significantly different LI of spindle density for patients with left and right focal epilepsy, with lower density in the epileptic hemisphere (****P < 0.0001, Fig. 4A; Supplementary Table 2). Furthermore, the raw density per hemisphere was close to the density reported by Purcell *et al.*^49^ and significantly lower in the epileptic hemisphere in comparison to the non-epileptic hemisphere (median, IQ in epileptic hemisphere: 1.40/min, 1.10–1.81/min and in the non-epileptic hemisphere: 1.61/min, 1.33–1.91/min, Wilcoxon test, *P* < 0.0001, Supplementary Fig. 2D). We also observed a significantly different LI of spindle amplitude in the opposite sense with higher values in the epileptic hemisphere (*P = 0.0402, Fig. 4B; Supplementary Table 2).

We did not find any significant LI differences between patients with right and left focal epilepsy when analysing slow wave density and duration (Fig. 3D and E) nor for duration of spindles and their locking to slow activity (Fig. 4C and D).

### Physiological sleep features in focal epilepsy are stable across two successive nights

Sleep-related activities can be affected by a ‘first-night effect’ when subjects sleep a first night in a new environment.^26^ We compared all sleep features between the two first consecutive nights in the EEG monitoring unit and within the groups of left and right focal epilepsy. We did not find any difference between consecutive nights for delta power (Supplementary Fig. 4) or any property of slow waves (Supplementary Fig. 5) or spindles (Supplementary Fig. 6).

### Physiological sleep features classify patients with left and right focal epilepsy

The LI of spindle density and delta power were among the physiological sleep parameters that most strongly discriminated left and right epilepsy, as illustrated in Fig. 5A and B. Mean accuracy, i.e. the proportion of correctly classified patients, reached 65% (SD: ± 5%, blue, right-sided line in Fig. 5C), which was significantly greater than accuracy obtained from shuffled data (50%, ± 7%, black, left-sided line in Fig. 5C and D, *P* < 0.0001, ANOVA + Sidak’s multiple comparison test). For the classification of left focal epilepsy, we obtained a sensitivity of 0.67, specificity of 0.63, positive predictive value of 0.63 and negative predictive value of 0.67. The values are simply inverted for right focal epilepsies. Statistical analysis on the contingency matrix also confirmed that the classifier performs above chance level (*P* = 0.018, Fisher’s exact test).

### Sleep features improve classification of epileptic lateralization based on IEDs

Having established that EEG signatures of physiological sleep can classify lateralization of epilepsy, we compared their classification accuracy against the one of IEDs. As expected, we observed a significantly different LI for IEDs between patients with left and right focal epilepsy (****P < 0.0001, Mann–Whitney test, Supplementary Fig. 7). Furthermore, we found a synergy between IEDs and physiological sleep features, as the accuracy of classification using a combination of both (77%, SD = 4%) was significantly higher than with sleep activities or IEDs alone (65%, SD = 5% and 75%, SD = 3%, respectively, both comparisons at *P* < 0.0001, Fig. 5D, ANOVA + Sidak’s multiple comparison test; see also Supplementary Fig. 8 for an example of a decision tree that used IEDs and sleep parameters in the classification algorithm). Hence, we found a small but significant increase in classification accuracy when combining sleep markers to IEDs, although IEDs were the most important parameter in classifying patients (Supplementary Fig. 9A). Next, we thus wanted to test whether the contribution of sleep markers surpassed the one of IEDs in ‘difficult-to-lateralize’ patients, i.e. those with a low asymmetry of IEDs rate. We measured the contribution of each parameter in the classification of the subset of patients whose LI of IEDs was below the absolute median LI of IEDs and observed that in this population, the importance of spindles density in classifying patients overtook the one of IEDs (Kruskal–Wallis + Dunn’s multiple comparison tests, *P* < 0.0001, Supplementary Fig. 9B).

## Discussion

### Interactions between sleep and focal epilepsy

Our study provides a multi-dimensional characterization of the cardinal EEG sleep features in patients with focal epilepsy. We show that certain features of typical EEG sleep markers are asymmetrically expressed in patients with focal epilepsy, and these asymmetries are stable across two consecutive nights. Our results suggest that during sleep, the underlying epileptic process not only manifests by IEDs but also influences physiological sleep activity. Our observations dovetail with the well-known modulation^32,36^ and in part lateralization^26,31,37,38^ of sleep activity by physiological neural processes induced by task or context (e.g. memory and somatosensory paradigms, first-night effect) and reveals that pathological processes such as epilepsy can imprint comparable shifts in the expression pattern of hypnic activities.

While other works have also shown an asymmetry of slow oscillations^23^ or spindles density^20^ in patients with epilepsy, conflicting evidence remains regarding spindles density.^21^ Furthermore, in this particular population, the asymmetric impact of a first-night effect^26^ on slow oscillations, described in healthy controls, has not been studied. Finally, our work provides also a more detailed analysis of sleep in epilepsy by including all the main sleep electrophysiological markers, in a significantly larger population and using a classifier that validates, on a patient-by-patient basis, observations that were only made on population-based analyses so far.

### Asymmetry of delta power and slow wave amplitude: a marker of increased sleep homeostasis?

We showed that delta power (Fig. 2) and slow wave amplitude (Fig. 3C), both related to slow wave activity,^23^ are asymmetrical in patients with focal epilepsy, with higher delta power and slow wave amplitude on the side of the epileptic hemisphere. In a previous study, several observations suggested that this increase in slow wave activity reflects a homeostatically driven mechanism: a correlation between the power of slow waves and the number of secondary generalized seizures in the previous 3–5 days, the lack of increase of slow wave activity during rapid-eye movement sleep, the similar locking of spindles to slow oscillations between controls and patients and the expected and physiological decline of slow wave activity along the night.^23^ Our larger cohort confirms the asymmetry of slow wave activity in patients with focal epilepsy.

Another interesting study showed that when healthy subjects sleep a first night in a novel environment, delta power in the left hemispheric default-mode network is decreased.^26^ Since this asymmetry is normalized during the second night, the authors interpreted this first-night effect as an expression of covert vigilance maintained by the left hemisphere in the context of sleeping in an unusual environment.^26^ We studied this putative effect of vigilance during the first night (expected to lead to decreased delta power in the left hemisphere^26^) in patients with left focal epilepsy, where homeostasis conversely translates into increased delta power in the left hemisphere.^23^ The lack of such a first-night effect in our study (Supplementary Fig. 4) could be due to higher homeostatic pressure in patients with epilepsy that overrules a more subtle physiological effect observed in healthy subjects. Alternatively, this lack of a first-night effect might be due to the fact that our low-density, surface EEG recordings sampled activity from a more widespread hemispheric network, while the effect in healthy subjects was seen in the default-mode network studied with magnetic source imaging.^26^ A third possibility could be that, in patients with left focal epilepsy, the neural processes related to vigilance would be shifted to the opposite hemisphere, thus avoiding interference from epileptic activity. However, the lack of a difference in lateralization between the first and second night in our study speaks against this hypothesis. While our work confirms the finding of increased slow wave activity in the epileptic hemisphere, further studies are thus needed to clarify the interaction between the homeostatic^23^ and vigilance^26^ mechanisms in patients with focal epilepsy.

### Asymmetry of slow wave slope: a marker of pathological synchronization in the epileptic hemisphere?

The finding of inter-hemispheric asymmetry of slow wave slope (Fig. 3F) sheds light on the ongoing neuronal dynamics that operate in the epileptic hemisphere. It is not surprising that slow wave amplitude (Fig. 3C) and slope (Fig. 3F) show the same asymmetry, as both are correlated with neuronal synchrony.^50^ Both synchronization and desynchronization have been described as potential pro-epileptogenic patterns.^51–54^ Here, the higher slope in the epileptic hemisphere reflects higher synchronization,^55^ which could play a major role in the expression of epilepsy. This is in line with the observation that IEDs are preceded by increased coupling between neuronal activity (as estimated by high-gamma activity) and slow oscillations.^11^ Indeed, this increased coupling represents a concentration, i.e. synchronization, of neuronal activity to a specific phase of the underlying slow oscillation. Thus, higher synchronization of neuronal activity in the epileptic hemisphere, suggested by our current study, could be reflected in the periodic increase of neuronal synchronization that anticipates IEDs in the irritative zone.^11^

### Asymmetry of spindle density and amplitude: a marker of the pathologic hemisphere?

Spindles are commonly considered to be symmetrical in adults but several studies have shown that they can segregate in space.^27,28,36,37^ We observed lower spindle density in the epileptic hemisphere (Fig. 4A). This observation is in line with previous publications,^20^ including the observation that spindles are less frequent before seizures,^17^ that their density is negatively correlated with IED density^18^ and that, in children with epilepsy, they tend to be localized remotely from the epileptic focus, i.e. in other lobes and in the opposite hemisphere.^19^ In contrast, another study observed higher spindle density in the epileptic hemisphere,^21^ but methodological differences could contribute to this discrepancy. First, that study used a spindle detector based on frequency power rather than detection of discrete events.^21^ Thus, abnormal activity in the same frequency range as spindles might have affected the results. Furthermore, analyses were performed on sleep-deprived patients, which is known to alter spindle density—albeit not necessarily asymmetrically.^56^ Of note, spindle density measured in our study (median, IQ: 1.55, 1.23–1.84/min) was in the range of values obtained by intracranial recording (median and IQ in Andrillon *et al.:*^28^ 1.3, 1.13–1.8/min), hence supporting accuracy of our technique for identifying spindles.

Our findings not only support and clarify discrepancies in the literature regarding spindle rate in focal epilepsy^20,21^ but also provide an extensive evaluation of the other main sleep electrophysiological hallmarks. Besides spindle rate, we also observed increased spindle amplitude on the epileptogenic side, which could relate to disproportionate synchronization.^44^

### Clinical perspectives: sleep as a surrogate biomarker of epilepsy

We showed that sleep activities are shaped by the underlying epileptic process, similarly to IEDs. Furthermore, we observed a slight but significant increase in classification accuracy when combining sleep activities to IEDs. This does not suggest that sleep parameters could replace IEDs, since sleep markers are only slightly improving the classification based on IEDs. However, sleep markers could become decisive when the analysis of IEDs is inconclusive. This is supported by the greater contribution of spindle density in the classification of patients with low asymmetry of IEDs (Supplementary Fig. 9) and should be further tested in larger cohorts. The current data suggest that the analysis of sleep markers could become a complementary measure in pre-surgical evaluation of epilepsy. Indeed, insight into hemispheric lateralization can be decisive in difficult-to-lateralize epilepsy, in which ambiguous, non-invasive pre-surgical planning can lead to the necessity of implanting bilateral intracranial electrodes or insufficient intracranial sampling leading to misleading results.^57,58^ This is particularly true in patients with temporal lobe epilepsy, in whom fast propagation of epileptic activity or bilateral independent discharges can lead to unclear lateralization.^59^ More generally, it is known that EEG has a low sensitivity to diagnose epilepsy, ranging from 25% to 56%.^60^ Even long-term EEG monitoring identifies IEDs in only 57% of patients with epilepsy who had previously normal EEG,^61^ which altogether calls for new biomarkers to be identified.

Future studies comparing hypnic activities in patients with various forms of epilepsy versus healthy controls will reveal whether asymmetries of physiological sleep could be used as an additional diagnostic tool. This could be particularly useful in the case of a first seizure, when there is no visible epileptiform activity on the EEG.

### Methodological considerations

From a methodological point of view, we chose to use the LI as the main read-out because this measure captures inter-hemispheric asymmetry of the sleep activity features on a patient-per-patient basis. Conversely, the absolute values of medians do not provide information about the distribution of individual values. Therefore, the LI appears more reliable than absolute medians to interpret our observations. Another methodological consideration is the choice of the reference. Surface EEG activity, and in particular slow activity, is usually assessed with an average reference. However, in the present case, it was important to identify activities as focally as possible, which motivated the bipolar montage.

We did not test collinearity between sleep markers and IEDs since the classifier is not forced to select all parameters to design a decision algorithm. If two parameters are highly correlated, the classifier may not select one of them in the decision algorithm (see Supplementary Fig. 8 for an example of a tree where only IEDs, spindle density and spindle amplitude are used). However, since the classification based on the combination of sleep markers and IEDs is slightly but significantly better than IEDs alone, this means that sleep markers contribute to the classification even when IEDs are included in the prediction (Supplementary Figs. 8 and 9).

Another limitation of our work is that we could not define whether the observed asymmetries stem from an actual decrease or increase compared to EEG activity in healthy subjects. We hence based our interpretations on comparison with findings from a corpus of previous publications.^4,17,18,23,26,62–64^ Given the variability of certain sleep-related activities, such as spindle density and amplitude,^44,49^ much larger cohorts, including healthy subjects, will be necessary to decipher how the asymmetry in patients with epilepsy differs from healthy controls.

## Conclusions

Our study shows that the fine inter-hemispheric balance controlling the expression of physiological sleep components on EEG is perturbed in the presence of lateralized epileptic activity. This effect is sufficiently strong to classify patients with right and left focal epilepsy, confirming that population differences still hold on patient-by-patient analyses. This raises important questions regarding the mechanisms governing the modulation of sleep markers by the underlying epileptic process.

## Supplementary Material

fcad161_Supplementary_Data

## Data Availability

Data are available upon reasonable request to the corresponding author.

## Abbreviations

IEDs =: interictal epileptiform discharges
ITC =: inter-trial coherence
IQ =: interquartile range
LI =: lateralization index
NREM =: non-rapid eye movement sleep
SD =: standard deviation
