# Supplementary material for: Asymmetry of sleep electrophysiological markers in patients with focal epilepsy
Source: Brain Commun. 2023 May 24;5(3):fcad161. doi: 10.1093/braincomms/fcad161 (PMC10244064; doi:10.1093/braincomms/fcad161)
Supplement: fcad161_Supplementary_Data [file fcad161_supplementary_data.docx]

**Supplementary Materials and Methods**

**Asymmetry of sleep electrophysiological markers in patients with focal epilepsy**

**Supplementary Figures and Tables**


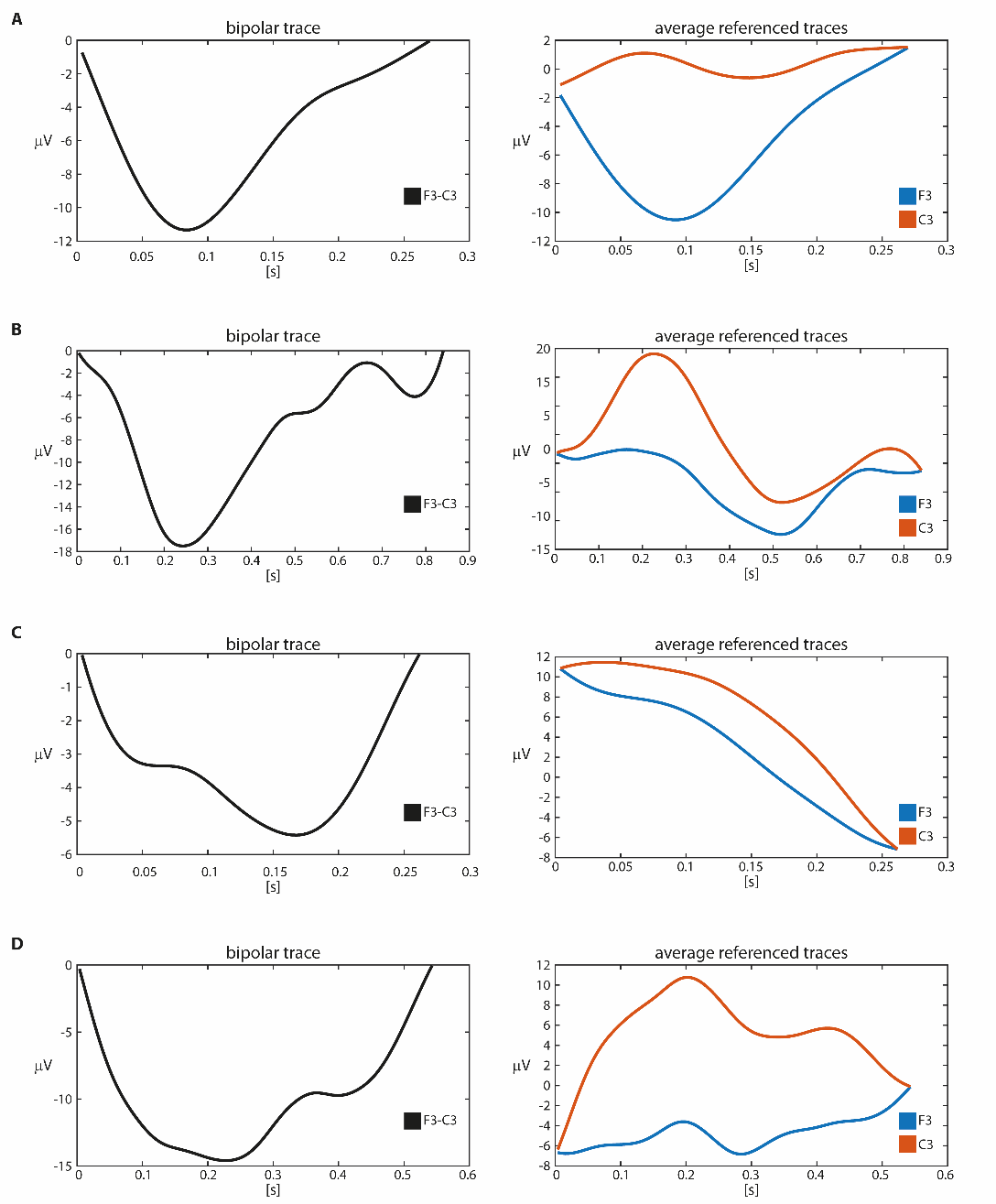


**Supplementary Figure 1 | Slow waves detection**

For each panel, the left graph is an example of a detected slow wave in the bipolar montage (F3-C3) and the right graph shows the corresponding signal in individual, average referenced electrodes (F3 and C3). **(A)** Example of a saved slow wave. **(B-D)** Examples of discarded slow waves. In **(B)**, the slow wave is discarded because, although both average referenced signals show a negative peak (around 0.5 s), the average referenced signal with the higher absolute value at the slow wave peak identified in the bipolar montage is positive. In **(C)**, the slow wave is discarded because no negative peak in the average referenced signal is identified during the duration of the slow wave identified in the bipolar montage. In **(D)**, the slow wave is discarded because, although one average referenced signal shows a negative peak along the duration of the slow wave (around 0.3 s), the signal with the highest positive value at the slow wave peak is positive.


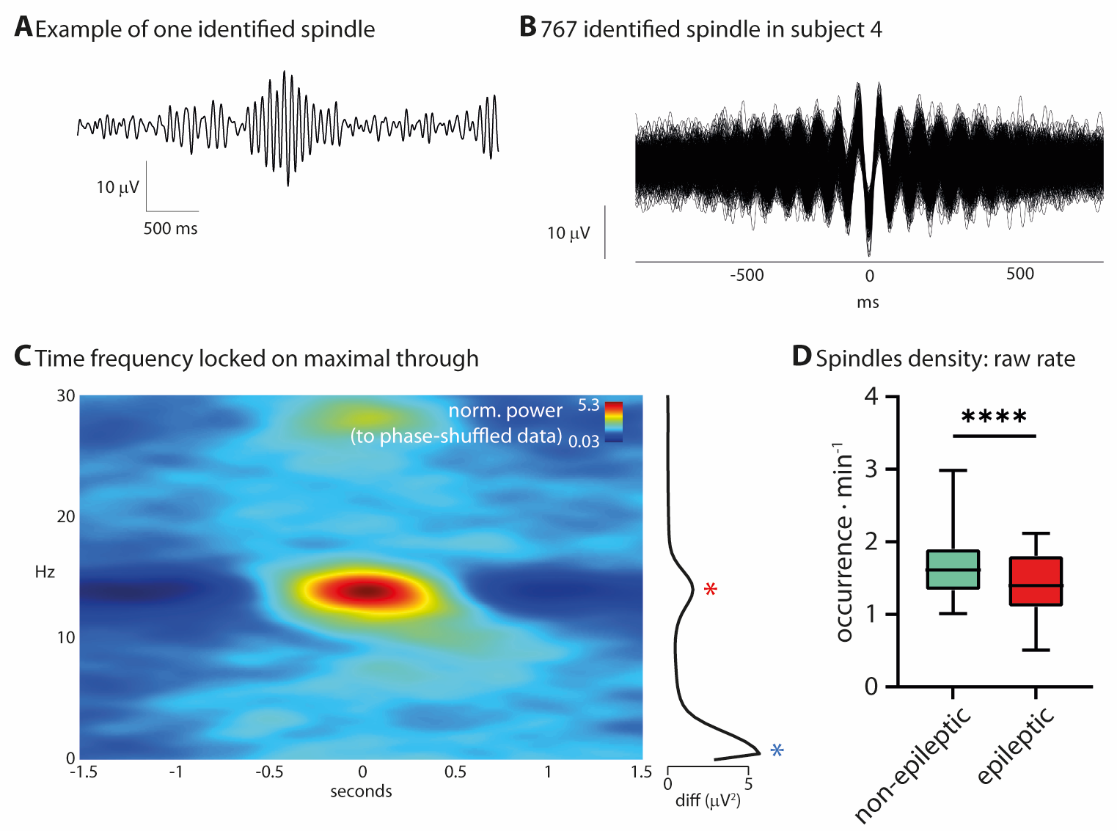


**Supplementary Figure 2 | Spindles identification**

**(A)** Example of one recorded spindle in subject 4 (filtered between 11-16 Hz). **(B)** Superposition of 767 spindles identified in subject 4, locked to the maximal trough (middle of the window) (filtered between 11-16 Hz). **(C)** Time-frequency analysis of the spindles depicted in B, normalized to phase-shuffled data (see Materials and Methods), showing a peak in the expected frequency range (14 Hz). The spectrogram on the right correspond to the oscillatory component of the data (see Material and Methods) between -0.5 to 0.5 s around the middle of the respective spindles’ time windows and shows, as expected, a peak in the spindle oscillatory frequency (14 Hz, red star) as well as an increased power in the slow oscillatory range (< 5 Hz, blue star), reflecting the occurrence of slow oscillations around spindles. **(D)** Occurrence of spindle per minute, per hemisphere. The non-epileptic hemisphere presents a significantly higher rate of spindles than the epileptic hemisphere (Wilcoxon test, *****p*<0.0001, n=69 patients).


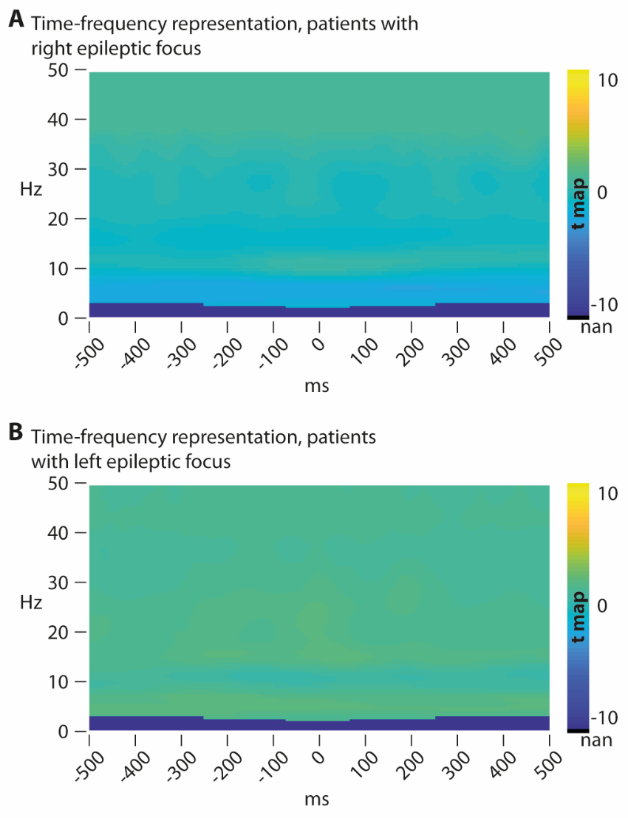


**Supplementary Figure 3 | Time-frequency decomposition locked to slow waves**

There is no significant difference of activity when comparing the time-frequency decomposition of the signal around (-500 to 500 ms, 0.1 to 50 Hz) slow waves in the epileptic vs non-epileptic hemisphere in patients with **(A)** right and **(B)** left focal epilepsy (cluster-based permutation test “Monte Carlo” method, as implemented in Fieldtrip ^1^). Since the size of the window of analysis is of 1 s, low frequencies cannot be assessed and are thus depicted in dark blue (note that they do not belong to the frequency of interest for this analysis, which is the one of interictal discharges, i.e., >15 Hz).


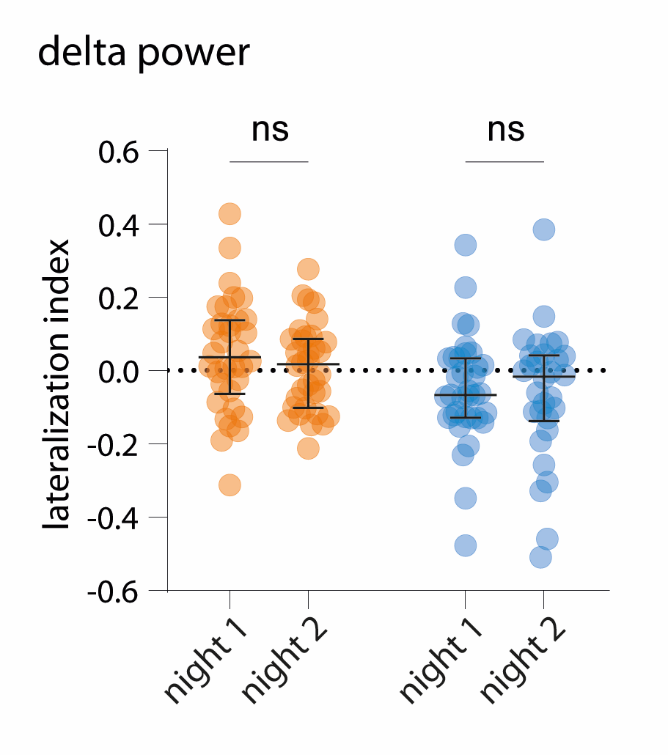


**Supplementary Figure 4 | Stability of delta power across two consecutive nights**

The comparison of the LI of delta power between the two first consecutive nights in the EEG monitoring unit within the groups of patients with left (orange) and right (blue) focal epilepsy did not show any difference (Wilcoxon test; n=34 patients with left focal epilepsy with available data for nights 1 and 2; n=30 patients with right focal epilepsy with available data for nights 1 and 2).

**
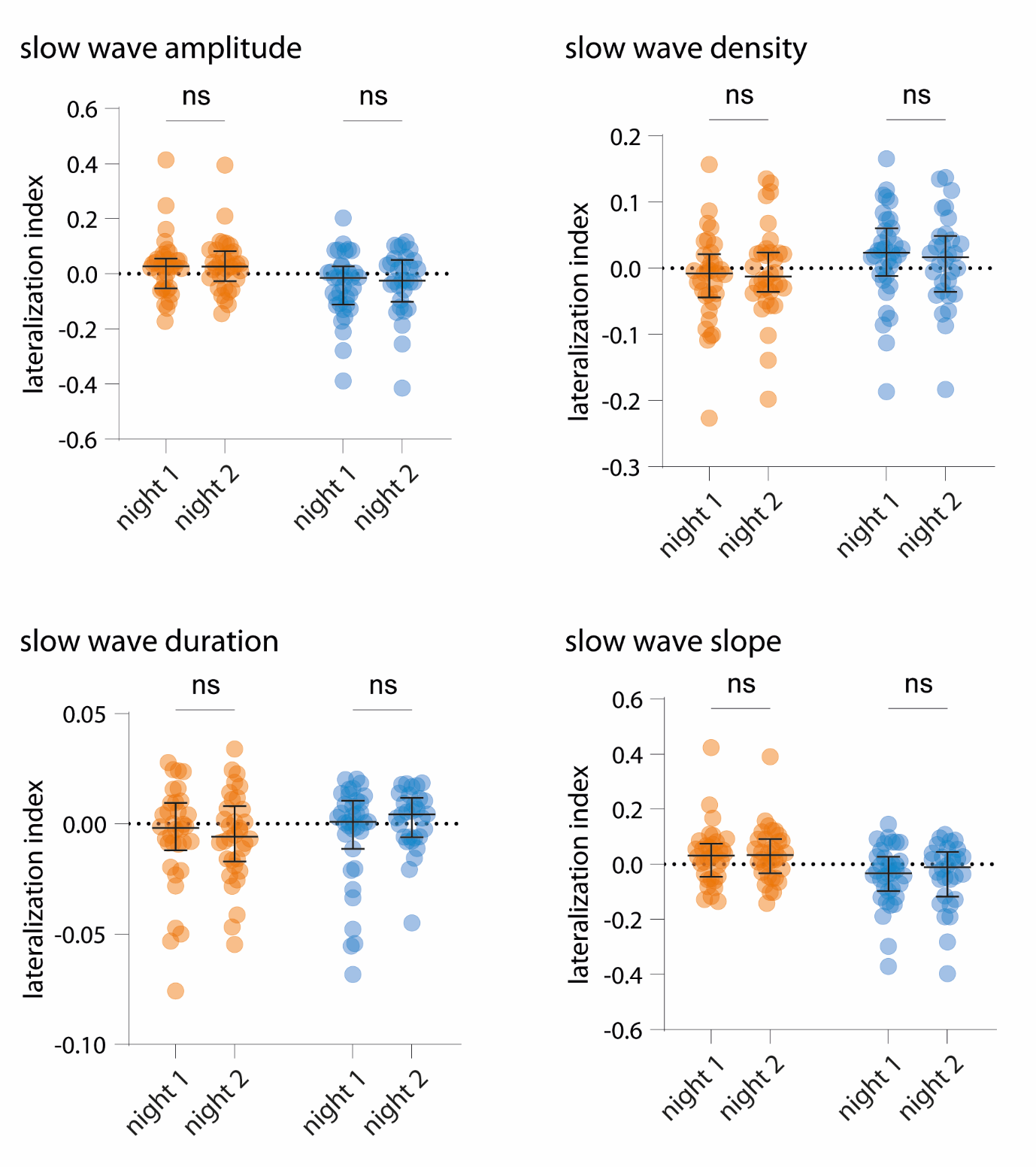
**

**Supplementary Figure 5 | Stability of slow wave properties across two consecutive nights**

The comparison of the LI of all slow wave properties between the two first consecutive nights in the EEG monitoring unit within the groups of patients with left (orange) and right (blue) focal epilepsy did not show any difference (Friedman test + Dunn’s correction for multiple comparisons ; n=34 patients with left focal epilepsy with available data for nights 1 and 2; n=30 patients with right focal epilepsy with available data for nights 1 and 2).

**
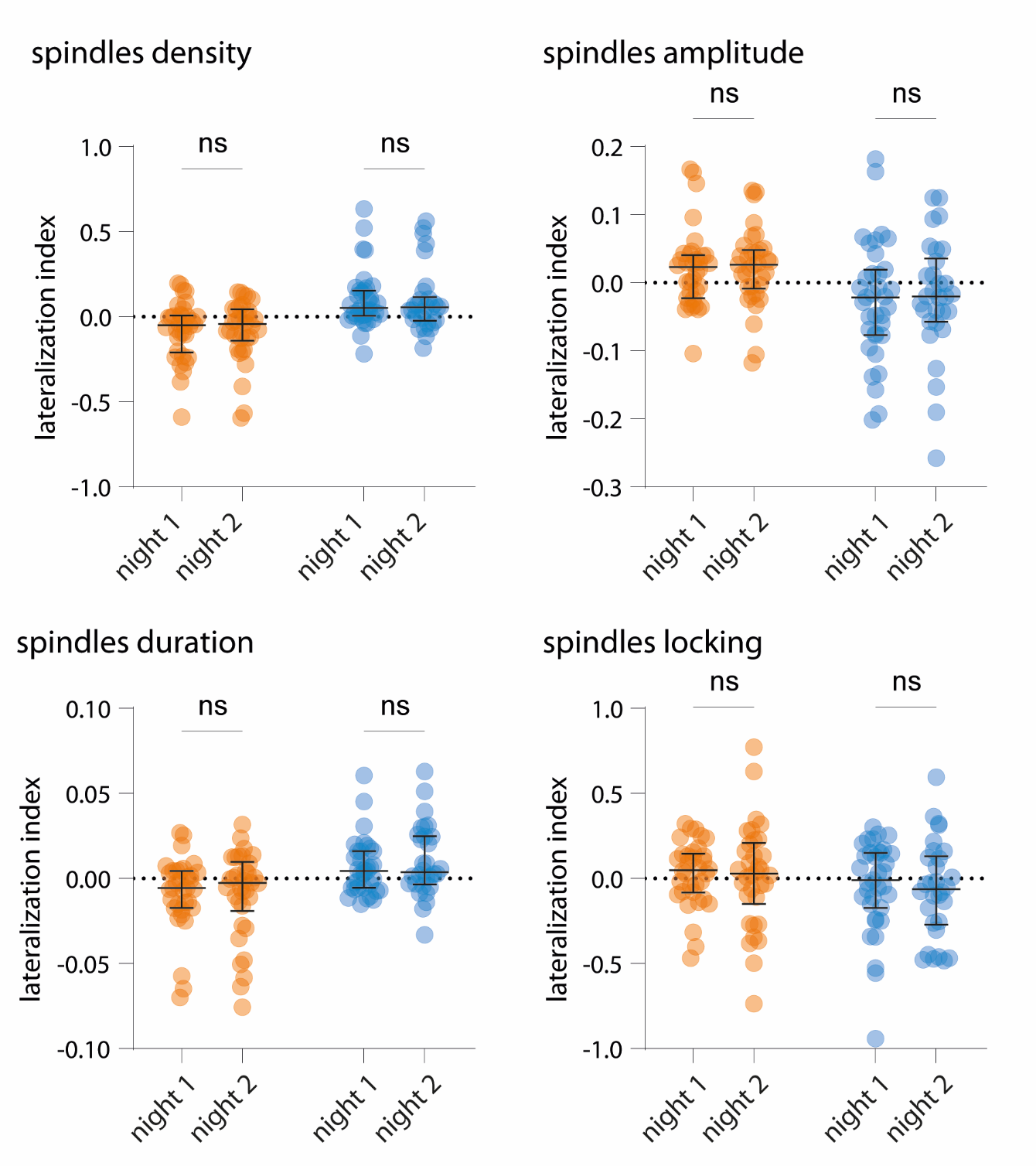
**

**Supplementary Figure 6 | Stability of spindle properties across two consecutive nights**

The comparison of the LI of spindle properties between the two first consecutive nights in the EEG monitoring unit within the groups of patients with left (orange) and right (blue) focal epilepsy did not show any difference (Friedman test + Dunn’s correction for multiple comparisons; n=34 patients with left focal epilepsy with available data for nights 1 and 2; n=30 patients with right focal epilepsy with available data for nights 1 and 2).

**
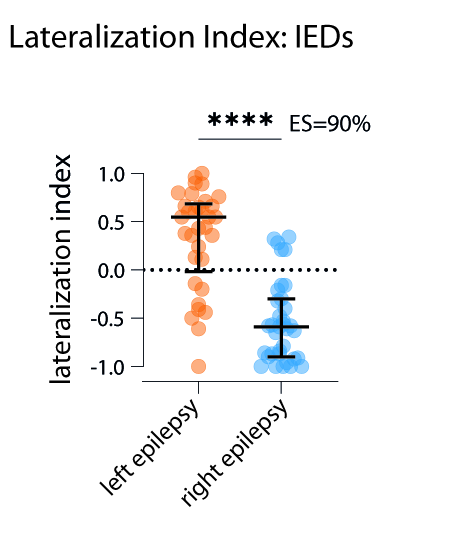
**

**Supplementary Figure 7 | Lateralization index of interictal epileptic discharges**

As expected, IEDs are highly asymmetric and more prevalent on the side of the epileptic focus (*****p*<0.0001, Mann-Whitney test, n=33 patients with left focal epilepsy and n=35 patients with right focal epilepsy).

Bars indicate median ± interquartile range. ES=non-parametric effect size (see Materials and Methods).

**
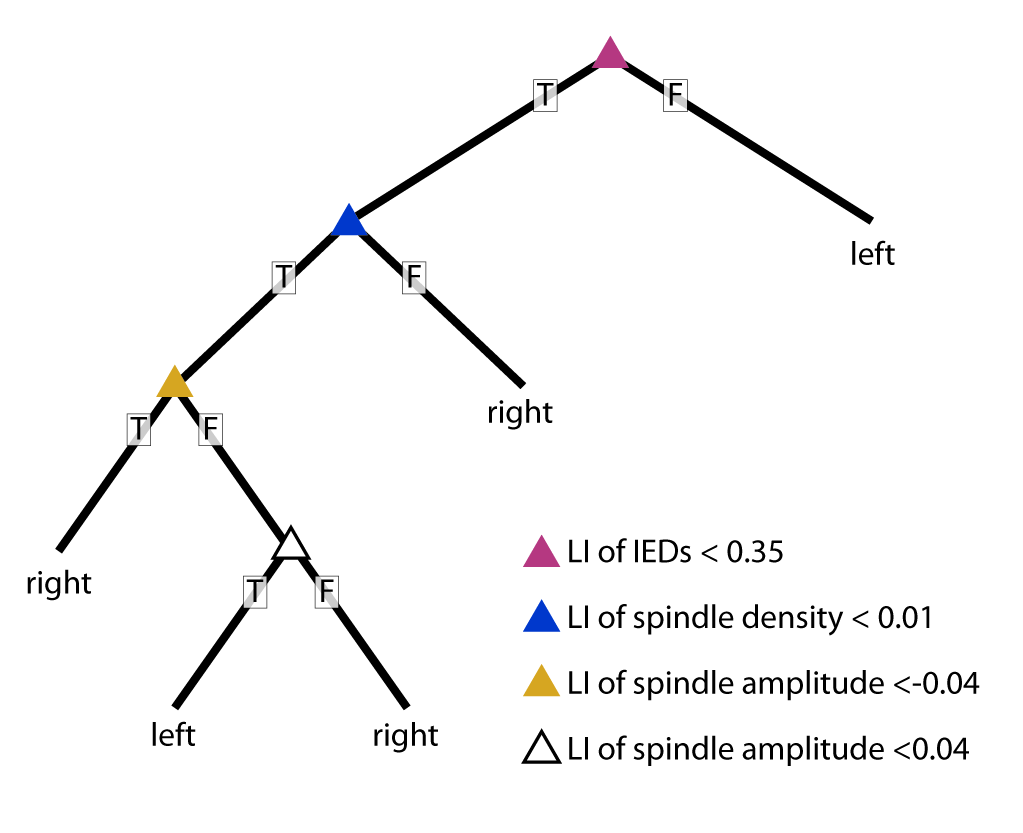
**

**Supplementary Figure 8 | Example of a decision tree**

This tree was extracted in the loop testing the accuracy of the sleep parameters and IEDs in classifying patients with left vs right focal epilepsy. Based on 3 items (IEDs, spindle density and spindle amplitude), it provided a classification with an accuracy of 86%. As exemplified here, all sleep parameters are not necessarily used by the classifier. T and F inside the white shaded boxes indicate True or False, which refer to the condition indicated by the colored triangles.

**
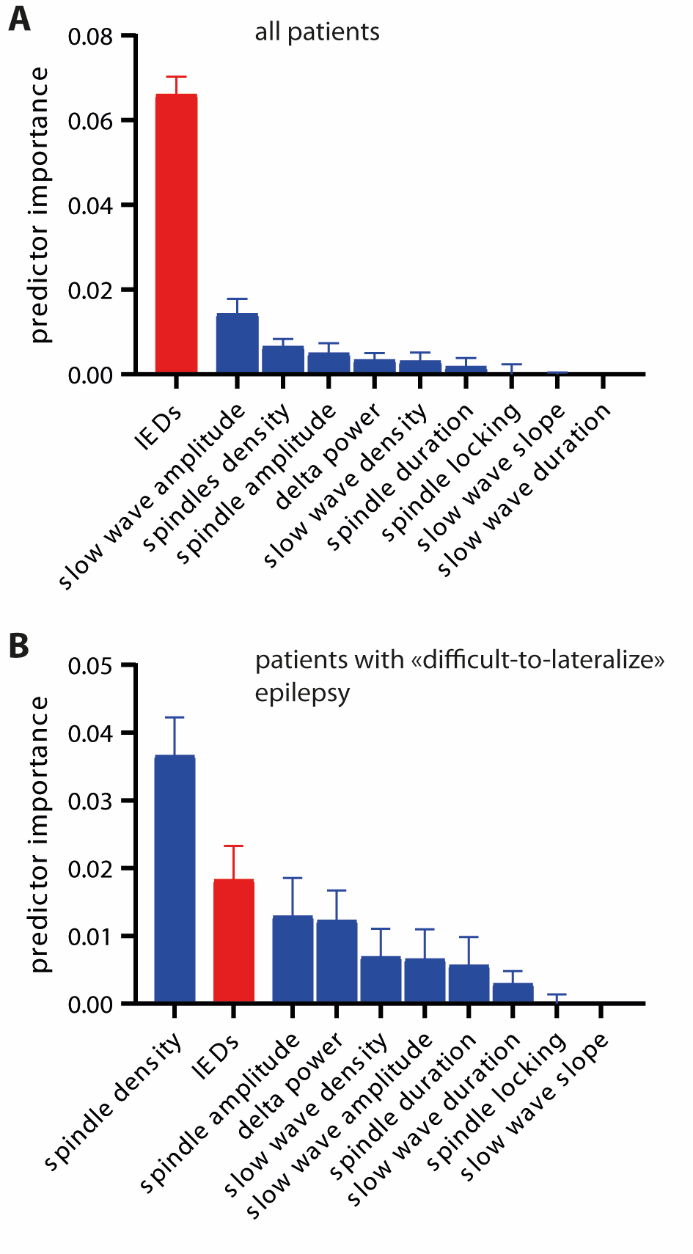
**

**Supplementary Figure 9 | Spindle density becomes more important than IEDs in classifying patients with difficult-to-lateralize epilepsy**

**(A)** Importance of each parameter for the classification of all 68 patients. As seen, IEDs dominate the contribution to classification (Kruskal-Wallis and correction for multiple comparisons of all sleep activities against IEDs: *p*<0.0001). **(B)** For “difficult-to-lateralize” epilepsies, i.e., patients whose Lateralization Index (LI) of IEDs is below the absolute median LI of IEDs, spindle density becomes significantly more important than IEDs in the classification of epileptic lateralization (Kruskal-Wallis and correction for multiple comparisons of all parameters against spindles density: *p*<0.0001).

**Supplementary Table 1 Population data**

| **Patient ID** | **Sex** | **Age at disease onset (Y)** | **Duration of the disease (M)** | **Age at EEG (Y)** | **Laterality** | **Lobar localisation** | **Etiology** | **Treatment** |
| --- | --- | --- | --- | --- | --- | --- | --- | --- |
| *1* | M | 52 | 111 | 61 | R | T | non lesional | LTG 200 mg 2x/j PGB 150 mg 2x/j |
| *2* | M | 16 | 163 | 29 | R | FT | non lesional | CBZ 400 mg 2x/j LEV 1000 mg 2x/j |
| *3* | M | 37 | 27 | 39 | R | hemispheric | post-traumatic | LTG 200 mg 2x/j LCM 200 mg 2x/j BRV 100 mg 2x/j |
| *4* | M | 16 | 117 | 26 | L | T | non lesional | none |
| *5* | M | 27 | 73 | 34 | R | P | dysplasia | LCM 200 mg 2x/j BRV 100 mg 2x/j PER 4 mg 1x/j PGB 150 mg 2x/j |
| *6* | M | 10 | 445 | 47 | R | F | non lesional | CBZ 400-0-800 LCM 200 mg 1x/j |
| *7* | M | 9 | 363 | 39 | L | T | post-traumatic | ZNS 400 mg 1x/j CBZ 400 mg 3x/j LTG 200 mg 2x/j |
| *8* | F | 15 | 102 | 23 | L | F | non lesional | LEV 1500-0-2000 ZNS 200 mg 1x/j |
| *9** | M | 18 | 31 | 20 | R | operculo-insular | dysplasia | LTG 50-0-100 |
| *10* | F | 25 | 424 | 60 | R | T | hippocampal sclerosis | LEV 1000 mg 2x/j |
| *11* | F | 23 | 103 | 32 | L | FT | non lesional | LEV 500 mg 2x/j LTG 300 mg 2x/j |
| *12* | M | 18 | 146 | 30 | L | F | non lesional | VPA 500 mg 2x/j PGB 200 mg 2x/j CLZ 0.25 mg 2x/j |
| *13* | M | 16 | 380 | 48 | R | F | non lesional | CLB 5-5-10 LTG 350-250-200 BRV 100 mg 2x/j |
| *14** | F | 12 | 144 | 24 | R | T | non lesional | LTG 100-0-300 |
| *15* | M | 22 | 48 | 27 | R | T | non lesional | OXC 300-0-900 |
| *16* | M | 11 | 88 | 18 | L | TO | non lesional | LTG 350 mg 2x/j CLB 10 mg 1x/j |
| *17* | F | 16 | 29 | 18 | R | Posterior quadrant | heterotopia | LTG 100 mg 2x/j PER 4 mg 1x/j ZNS 100 mg 1x/j BRV 100 mg 2x/j CLB 5 mg 2x/j |
| *18* | F | 33 | 113 | 42 | R | T | status post autoimmune encephalitis | LTG 200 mg 2x/j PER 8 mg 1x/j ZNS 300 mg 1x/j |
| *19* | M | 13 | 277 | 36 | L | FT | hippocampal sclerosis | LTG 300 mg 2x/j LEV 1000 mg 3x/j LCM 150 mg 2x/j CLZ 0.5 mg 1x/j |
| *20* | F | 15 | 224 | 33 | L | hemispheric | non lesional | PGB 100 mg 2x/j CBZ 600 mg 2x/j |
| *21* | M | 32 | 45 | 35 | L | F | dysplasia | VPA 500-0-1000 OXC 600 mg 2x/j |
| *22* | F | 4 | 157 | 17 | L | F | dysplasia | OXC 600 mg 2x/j |
| *23* | F | 26 | 163 | 39 | L | T | non lesional | LCM 200 mg 2x/j CLZ 1 mg 1x/j |
| *24* | M | 2 | 333 | 30 | L | TO | post-infectious | LTG 400-0-100 ZNS 200 mg 2x/d BRV 50 mg 2x/jd |
| *25* | M | 6 | 194 | 22 | L | PO | non lesional | LEV 500-0-1000 LCM 150 mg 2x/d TPX 200 mg 1x/d |
| *26* | M | 4 | 394 | 36 | R | T | hippocampal sclerosis | CLB 10 mg 2x/d VPA 750 mg 2x/d LTG 200 mg 2x/d |
| *27* | M | 2 | 591 | 51 | L | T | hippocampal sclerosis | CBZ 200 mg 2x/d LTG 200 mg 3x/d LCM 200-100 LEV 1000 mg 2x/d CLB 5 mg 1x/d |
| *28* | F | 37 | 2 | 38 | R | T | non lesional | LTG 100 mg 2x/d PGB 150 mg 2x/d |
| *29* | F | 21 | 323 | 48 | R | T | non lesional | PER 10 mg 1x/d LTG 150 mg 1x/d TPX 100-0-50 CLB 5 mg 1x/d |
| *30* | F | 37 | 34 | 40 | R | F | vascular | LTG 200-300-200 PER 6 mg 1x/d |
| *31* | M | 4 | 592 | 53 | L | T | hippocampal sclerosis | LTG 100 mg 2x/d VPA 500-1000 |
| *32* | F | 12 | 366 | 42 | R | TP | non lesional | LCM 200 mg 2x/d PER 4 mg 1x/d CLZ 0.5 mg 1x/d CLB 10 mg 1x/d |
| *33* | F | 14 | 39 | 17 | R | T | hippocampal sclerosis | LTG 300 mg 2x/d |
| *34* | F | 2 | 437 | 38 | R | P | dysplasia | LTG 300 mg 2x/d PER 6 mg 1x/d |
| *35* | M | 23 | 269 | 45 | L | cingulate | benign tumor | CBZ 600 mg 2x/d LTG 150 mg 1x/d |
| *36* | M | 34 | 64 | 39 | R | T | dysplasia | OXC 900-600-900 |
| *37* | M | 8 | 153 | 20 | L | T | post-infectious | LEV 1500 mg 2x/d OXC 750-0-900 |
| *38** | M | 17 | 68 | 23 | R | cingulate | non lesional | LTG 100 mg 2x/d LEV 500 mg 2x/d |
| *39* | M | 25 | 61 | 30 | R | F | non lesional | VPA 1000 mg 2x/d CLZ 0.25 mg 2x/d LCM 100 mg 2x/d |
| *40* | F | 11 | 82 | 17 | L | T | hippocampal sclerosis | LTG 150 mg 2x/d |
| *41* | M | 3 | 171 | 18 | L | T | hippocampal sclerosis | TPX 125-0-150 CBZ 750 mg 2x/d |
| *42* | F | 5 | 231 | 24 | R | F | dysplasia | TPX 450 mg 1x/d LCM 500 mg 1x/d |
| *43* | M | 33 | 198 | 49 | R | T | hippocampal sclerosis | VPA 1000 mg 2x/d LEV 1000 mg 2x/d |
| *44* | F | 6 | 318 | 32 | L | cingulate | non lesional | CBZ 600-0-400 LEV 500 mg 2x/d LCM 100 mg 2x/d |
| *45* | M | 24 | 179 | 39 | L | FT | post-traumatic | LTG 125-0-150 LCM 100 mg 2x/d |
| *46* | M | 20 | 45 | 24 | R | T | possible DNET | OXC 450-0-900 LTG 100 mg 2x/d |
| *47** | M | 13 | 221 | 31 | R | F | non lesional | LTG 200-0-300 LEV 750 mg 2x/d ETX 500 mg 3x/d |
| *48* | F | 18 | 12 | 19 | R | hemispheric | non lesional | LTG 200 mg 2x/d |
| *49* | F | 40 | 38 | 43 | L | T | encephalocele | LTG 350 mg 2x/d OXC 300-0-600 lorazepam 1 mg 1x/d |
| *50* | F | 25 | 47 | 29 | L | T | non lesional | LTG 200 mg 2x/d PGB 150 mg 2x/d |
| *51* | F | 20 | 341 | 48 | R | T | non lesional | LEV 1500 mg 2x/d LCM 200 mg 2x/d VPA 1200 mg 1x/d CLB 2.5 mg 1x/d |
| *52* | M | 16 | 17 | 17 | L | FT | non lesional | OXC 900 mg 2x/d LTG 400 mg 2x/d CLB 5 mg 4x/wk |
| *53* | F | 8 | 346 | 36 | L | TPO | perinatal injury | LTG 150 mg 2x/d |
| *54* | M | 18 | 50 | 22 | L | T | ganglioglioma | VPA 800 mg 2x/d |
| *55** | M | 23 | 9 | 24 | R | FT | non lesional | OXC 450 mg 2x/d LCM 100-0-200 CLZ 0.5 mg 1x/d |
| *56* | F | 16 | 57 | 21 | L | T | hippocampal sclerosis | LTG 250-0-200 |
| *57* | F | 45 | 47 | 49 | R | T | hippocampal sclerosis | LEV 500 mg 2x/d LTG 125 mg 2x/d lorazepam 2.5 mg 1x/d |
| *58* | M | 41 | 196 | 58 | L | FTP | vascular | LEV 1000 mg 2x/d LTG 200 mg 2x/d PB 150 mg 1x/d GBP 400 mg 3x/d |
| *59* | M | 14 | 119 | 24 | R | T | hippocampal sclerosis | BRV 100 mg 1x/d LTG 100 mg 1x/d |
| *60* | M | 13 | 118 | 22 | R | TP | heterotopia | LTG 200 mg 2x/d BRV 100 mg 2x/d PER 8 mg 1x/d |
| *61* | F | 6 | 141 | 18 | R | T | hippocampal sclerosis | OXC 750 mg 2x/d LTG 150 mg 2x/d |
| *62* | F | 3 | 271 | 25 | R | T | non lesional | LTG 200-0-100 LEV 500 mg 2x/d |
| *63* | M | 27 | 91 | 35 | L | FT | non lesional | LCM 150-0-200 PER 6 mg 1x/d |
| *64* | F | 12 | 463 | 50 | R | T | hippocampal sclerosis | LEV 500 mg 2x/d LCM 100 mg 2x/d CLZ 0.5 mg 1x/d |
| *65* | M | 46 | 82 | 52 | L | T | hippocampal sclerosis | CBZ 300 mg 2x/d |
| *66* | M | 24 | 161 | 37 | L | F | non lesional | LEV 2000 mg 2x/d LTG 200 mg 2x/d PER 8 mg 1x/d |
| *67* | F | 2 | 429 | 37 | L | FT | unclear, possibly hippocampal sclerosis | ZNS 300 mg 2x/d |
| *68* | M | 17 | 17 | 18 | L | T | non lesional | none |
| *69* | M | 37 | 60 | 42 | L | hemispheric | non lesional | PGB 100-0-150 LTG 300 mg 2x/d BRV 100-0-50 |

M: male, F: female, Y: years, M: months, R: right, L: left, T: temporal, F: frontal, FT: fronto-temporal, TO: temporo-occipital, TP: temporo-parietal, P: parietal, PO: parieto-occipital, TPO: temporo-parieto-occipital, FTP: fronto-temporo-parietal, BRV: brivaracetame, CBZ: carbamazepine, CLB: clobazam, CLZ: clonazepam, ETX: ethosuximide, GBP: gabapentine, LCM: lacosamide, LEV: levetiracetame, LTG: lamotrigine, OXC: oxcarbazepine, PB: phenobarbital, PER: perampanel, PGB: pregabaline, TPM: topiramate, VPA: valproate, ZNS: zonisamide. Benign tumors included ependymoma, meningioma and cavernoma. Patients marked with an * are not included in the second night because of intercurrent seizures during night 2 only and were thus not included in the comparisons between nights 1 and 2 (Supplementary Fig. 4-6).

**Supplementary Table 2 Details of statistical analyses**

| **Lateralization index:**  **median ± interquartile range** | **Right** | **Left** | ***p-val (corr.)*** | **Test** | **Figure** |
| --- | --- | --- | --- | --- | --- |
| **Delta power** | -0.05 (-0.13 to 0.005) | 0.02 (-0.05 to 0.10) | ***0.0081*** | Mann Whitney | **2A** |
| **Slow wave amplitude** | -0.03 (-0.11 to 0.03) | 0.03 (-0.04 to 0.07) | ***0.0158*** | Kruskal-Wallis | **3C** |
| **Slow wave density** | 0.02 (-0.01 to 0.06) | -0.01 (-0.04 to 0.02) | 0.1035 | Kruskal-Wallis | **3D** |
| **Slow wave duration** | 0.003 (-0.01 to 0.01) | -0.003 (-0.02 to 0.01) | >0.99 | Kruskal-Wallis | **3E** |
| **Slow wave slope** | -0.04 (-0.10 to 0.03) | 0.04 (-0.04 to 0.09) | ***0.0096*** | Kruskal-Wallis | **3F** |
| **Spindles density** | 0.04 (0.01 to 0.14) | -0.05 (-0.15 to 0.03) | ***<0.0001*** | Kruskal-Wallis | **4A** |
| **Spindles amplitude** | -0.02 (-0.07 to 0.03) | 0.03 (-0.01 to 0.05) | ***0.0402*** | Kruskal-Wallis | **4B** |
| **Spindles duration** | 0.003 (-0.005 to 0.02) | -0.004 (-0.02 to 0.005) | 0.6428 | Kruskal-Wallis | **4C** |
| **Spindles locking** | -0.07 (-0.16 to 0.06) | 0.004 (-0.09 to 0.15) | 0.0932 | Kruskal-Wallis | **4D** |

**Slow waves identification and processing**

For each slow wave identified in bipolar traces, based on published criteria^2,3^, we further verified whether a negative potential is present in either of the two average referenced signals involved in the dipole (e.g., F3 or C3):

- If both average referenced traces are positive at the negative peak of the slow wave identified in the bipolar montage, the slow wave is discarded
- If both average referenced traces show a negative peak at some point in the slow wave identified in the bipolar montage, the slow wave is saved
  - if both average referenced traces are negative at the moment of the negative peak identified in the bipolar montage or
  - if only one average referenced trace is negative at the moment of the negative peak identified in the bipolar montage, then this negative value should be higher, in absolute terms, than the positive value of the other average referenced trace (see Supplementary Fig. 1A for a saved and Supplementary Fig. 1B for a discarded slow wave, based on this criterion)
- If only one average referenced trace shows a negative peak at some point in the slow wave identified in the bipolar montage, the slow wave is saved if, at the moment of the negative peak identified in the bipolar montage, the negative value of that averaged referenced trace is higher, in absolute terms, than the other average referenced trace (see Supplementary Fig. 1D)
- If none of the average referenced trace show a negative peak at some point in the slow wave identified in the bipolar montage, the slow wave is discarded (see Supplementary Fig. 1C).

Supplementary Fig. 1 shows typical examples of saved and discarded slow waves. This automatic step was a necessary compromise between the requirement to identify focal events and the concern to identify negative waves using bipolar traces in order to make valid inferences with the published literature.^4,5^

Once slow waves were identified, we kept only those with amplitude >25^th^ percentile along all detected slow waves within patient and hemisphere. This was chosen to follow the amplitude criteria of Frauscher et al.^2^. Although in this paper^2^ the authors selected slow waves with amplitude >75^th^ percentile, we lowered that threshold to prevent a saturation effect, since we wanted to test potential differences of amplitude between hemispheres.

To verify that the observations related to slow waves were not contaminated by intercurrent interictal epileptic discharges including slow activity, we performed the following analysis. We extracted blindly ~1000 epochs around slow waves (-2 s before onset and +2 s after offset) for each patient and compared the time-frequency decomposition between hemispheres (Supplementary Fig. 3). For the time-frequency analyses, we used a wavelet decomposition with a sliding window equal to three cycles of each individual frequency from 0.1 to 50 Hz (*ft_freqanalysis.m*). For the statistical analysis, we performed a paired t-test (method: montecarlo; number of randomizations: 1000, correction for multiple comparisons: cluster) from -500 to 500 ms and 0.1 to 50 Hz around the detected onset of slow waves (*ft_freqstatistics.m*).

**Classification based on sleep features: lateralization index of IEDs**

To obtain the lateralization index of IEDs, we analysed the data with Epilog® NV (Ghent, Belgium), which uses an automatic detector of IEDs developed by Persyst®. The detection algorithm proved to be non-inferior to humans for spike detection.^6^ We calculated the LI between the number of epileptic discharges identified in left hemispheric electrodes and right hemispheric electrodes (removing the midline electrodes, Supplementary Fig. 7). The detection was not available in one patient and the analysis was thus performed on the remaining 68 patients. Given that visual interpretation of IEDs by expert epileptologists is part of the definition of our gold standard of epileptic lateralization, we relied on the automatic detection of IEDs to determine the ability of IEDs to lateralise the epileptic hemisphere, in order to avoid user-related bias and circularity.

**Supplementary references**

1. Oostenveld R, Fries P, Maris E, Schoffelen JM. FieldTrip: Open Source Software for Advanced Analysis of MEG, EEG, and Invasive Electrophysiological Data. *Computational Intelligence and Neuroscience*. 2011;2011:1-9. doi:10.1155/2011/156869

2. Frauscher B, von Ellenrieder N, Ferrari-Marinho T, Avoli M, Dubeau F, Gotman J. Facilitation of epileptic activity during sleep is mediated by high amplitude slow waves. *Brain*. 2015;138(6):1629-1641. doi:10.1093/brain/awv073

3. Riedner BA, Vyazovskiy VV, Huber R, et al. Sleep Homeostasis and Cortical Synchronization: III. A High-Density EEG Study of Sleep Slow Waves in Humans. *Sleep*. 2007;30(12):1643-1657. doi:10.1093/sleep/30.12.1643

4. Nir Y, Staba RJ, Andrillon T, et al. Regional Slow Waves and Spindles in Human Sleep. *Neuron*. 2011;70(1):153-169. doi:10.1016/j.neuron.2011.02.043

5. Massimini M, Huber R, Ferrarelli F, Hill S, Tononi G. The Sleep Slow Oscillation as a Traveling Wave. *Journal of Neuroscience*. 2004;24(31):6862-6870. doi:10.1523/JNEUROSCI.1318-04.2004

6. Scheuer ML, Bagic A, Wilson SB. Spike detection: Inter-reader agreement and a statistical Turing test on a large data set. *Clinical Neurophysiology*. 2017;128(1):243-250. doi:10.1016/j.clinph.2016.11.005
